# Gastrointestinal Stromal Tumors: 10-Year Experience in Cancer Center—The Ottawa Hospital (TOH)

**DOI:** 10.3390/curroncol29100562

**Published:** 2022-09-29

**Authors:** Abdulhameed Alfagih, Abdulaziz AlJassim, Bader Alshamsan, Nasser Alqahtani, Timothy Asmis

**Affiliations:** 1 Division of Medical Oncology, Department of Medicine, The Ottawa Hospital, The University of Ottawa, Ottawa, ON K1H 8L6, Canada; 2 Medical Oncology Department, Comprehensive Cancer Center, King Fahad Medical City, Riyadh 11525, Saudi Arabia; 3 Department of Medicine, College of Medicine, Qassim University, Qassim 51452, Saudi Arabia; 4 King Abdulaziz Hospital, Ministry of National Guard Health Affairs, Al Ahsa 36427, Saudi Arabia

**Keywords:** gastrointestinal stromal tumors, GIST, C-Kit, imatinib mesylate, survival

## Abstract

(1) Background: The management of gastrointestinal stromal tumors (GIST) has significantly evolved over the last two decades, with the introduction of tyrosine kinase inhibitors (TKI). We aim to report 10 years of experience of GIST management at a regional cancer center in Canada. (2) Methods: We retrospectively analyzed the records of 248 consecutive patients diagnosed with GIST between 2011 and 2021. We describe the clinical and pathological data, management, and outcome, including survival. (3) Results: The most common GIST sites were the stomach 63% (156), followed by the small bowel 29% (73). At diagnosis, 83% (206) of patients had localized disease (stage I–III). According to the modified National Institutes of Health consensus criteria (NIH) for GIST, around 45% (90) had intermediate or high-risk disease. Most patients, 86% (213), underwent curative surgical resection. Forty-nine patients received adjuvant imatinib, while forty-three patients had advanced disease and received at least one line of TKI. With a median follow-up of 47 months, the 5-year recurrence-free survival (RFS) rates for very low and low risk were 100% and 94%, respectively, while those for intermediate and high risk were 84% and 51%, respectively. The 5-year overall survival (OS) rates for very low and low risk were 100% and 94%, while intermediate, high risk, and advanced were 91%, 88%, and 65%, respectively. Using the Kaplan–Meier method, there were statistically significant differences in RFS and OS between NIH risk groups, *p* < 0.0005. In univariate analysis, ECOG, site, mitosis, secondary malignancy, and size were predictors for OS. High mitosis and large size (>5 cm) were associated with worse RFS. (4) Conclusions: Curative surgical resection remains the gold standard management of GIST. Our results are comparable to the reported literature. Further research is needed to explore histology’s role in risk stratification and initiating adjuvant TKI.

## 1. Introduction

Gastrointestinal stromal tumors (GIST) are rare non-epithelial tumors derived from mesenchymal tissues. They originate from interstitial Cajal cells or the stem cell precursors of these cells, located around the myenteric plexus throughout the gastrointestinal tract [1,2]. GIST account for 1–3% of all GI tumors. In 1983, two pathologists, Mazur and Clark, introduced the term GIST. Subsequently, further research led to a considerable understanding of the pathogenesis and biology of this type of tumor [3]. Identifying c-KIT mutation was a breakthrough that allowed for better characterization and identification of GIST, based on molecular studies. Further studies of the c-KIT pathway identified many important mutations with clinical and therapeutic significance, including exons 11, 9, 13, and 17 and platelet-derived growth factor receptor alpha gene (PDGFRA) [4,5]. 

Surgical resection is the standard treatment for localized GIST, while debulking surgery may be considered for symptomatic advanced bulky tumors. For small asymptomatic GIST, watchful waiting may be a reasonable option. The introduction of imatinib, a selective inhibitor of the KIT protein tyrosine kinase, in the management of GIST, revolutionized the treatment approaches [6,7]. Adjuvant imatinib has substantially improved recurrence-free survival in many phase III trials, particularly in the intermediate and high-risk groups [8].

In advanced GIST, many TKIs are shown to improve PFS and OS. In KIT-positive GISTs, imatinib is considered the standard first-line treatment [9]. Resistance due to secondary KIT mutations is expected despite the remarkable responses achieved with imatinib [10]. Sunitinib is an appropriate second-line option for patients who progressed on imatinib or had intolerance or resistance to imatinib [11]. Regorafenib improved PFS, when used after imatinib, and sunitinib failure, based on the phase III GRID trial result [12]. Ripretinib, as a fourth-line therapy, showed improvement in PFS over a placebo in the INVICTUS trial. Many evolving options have improved PFS, based on phase II trials, such as Sorafenib, nilotinib, avapritinib, and dasatinib [13,14,15,16,17]. 

The management of GIST has had a significant evolution over the last two decades. The data from real world practice from Canada on GIST are limited. Our study aims to report a 10-year experience in the management of GISTs in a regional cancer Centre in Canada- The Ottawa Hospital (TOH).

## 2. Materials and Methods

This is an observational retrospective cohort study. We identified GIST cases by searching hospital databases using ICD 10 codes. We examined records of all GIST cases referred to/or diagnosed in TOH between 1 January 2011 and 31 December 2021. Only patients with biopsy-proven and immunohistochemistry-confirmed diagnoses of GIST were included in the study. Details of the tumor site, risk assessments, and clinical, pathological, management, and outcomes data were recorded.

Risk assessment was estimated according to modified NIH and Miettinen risk criteria. Data were collected from the electronic medical records (Epic) on the access database. Results were analyzed using MS Excel and SPSS 25.0 software. Descriptive statistics are used to summarize data and synthesize and report patients’ demographic and clinicopathological data. Qualitative variables were analyzed by χ^2^ test and Fisher’s exact test. Survival data (RFS, PFS, OS) were analyzed using Kaplan–Meier methods and compared by log-rank test. OS was calculated from the date of tissue diagnosis to the date of death or last follow-up. Recurrence-free survival (RFS) was calculated from the date of surgical intervention to the date of recurrence or death or last follow-up. PFS for imatinib was calculated from the date of starting imatinib to the date of confirmed progressive disease (including escalated dose) or death or last follow-up. Potential prognostic factors were analyzed using the Cox proportional hazard model for multivariate analysis. Two-tailed *p*-values were reported and were considered to be statistically significant when *p* < 0.05.

## 3. Results

### 3.1. Patient Characteristics

The patient’s’ characteristics are shown in Table 1. In total, 248 patients were identified with a median age of 64 (range 28–90); males and females were a 1:1 ratio. An average of 23 cases were diagnosed per year (range 16–32).

### 3.2. Sites 

Sites and pathology data are shown in Table 2. The most common GIST sites were the stomach (156 patients, 63%), followed by the small bowel (73 patients, 29%). Other sites, including the esophagus, appendix, colon and rectum, and mesentery, collectively comprise 8% (19). The duodenum and jejunum represent around 52% of small bowel GIST (*n* = 19, 26% for each), while the ileum represents 17% (13). 

### 3.3. Clinical Presentation

The most common presentation was an incidental finding during workup for another clinical problem, followed by abdominal pain, representing 35% (87) and 34% (84), respectively. GI bleeding and anemia were the main presenting symptoms, representing 24% (59) and 19% (48), respectively. Only 4% (10) of patients presented with bowel obstruction or perforation. Among those presenting with symptoms, 13% (32) had symptoms for more than six months. Around 17% (41) had acute symptoms (less than 14 days). In addition, 71% (177) of patients had an excellent performance status, ECOG 0 to 1. Three cases had familial GIST. A CT scan made the provisional diagnosis in around half of the cases. Moreover, 30% (76) were diagnosed by either endoscopy or EUS. Nearly 53% (131) had TNM stage I disease at presentation, while 13% (32) had stage IV disease. Using a chi-squared test, there was a statistically significant association between gender* TNM stage (χ^2^(4) = 10.4, *p* = 0.034) and gender* NIH risk class (χ^2^(6) = 13.5, *p* = 0.036). Males had more advanced and higher-risk disease compared to females.

### 3.4. Pathology Data

The majority, 64% (159), had spindle cell histology, followed by mixed and epithelioid histology, 16% (39) and 7% (18), respectively. Very small tumors (less than 2 cm) were found in 15% (37). At the same time, larger tumors of more than 10 cm were seen in 13% (31). The majority has a low mitosis rate—less than five mitoses per HPF, 71% (177). 

### 3.5. Risk Assessment 

According to NIH criteria for nonmetastatic GIST, 24% (48) and 21% (42) had intermediate or high-risk diseases. While using the Miettinen criteria, 19% (39) and 12% (24) had moderate or high-risk diseases.

### 3.6. Molecular Data

Mutation testing is not routinely done. Only 11 patients had mutational analysis. Among them, four have PDGFR mutation, and three have KIT exon 11 mutation. 

### 3.7. Management Data and TKI Use 

**Figure 1:**
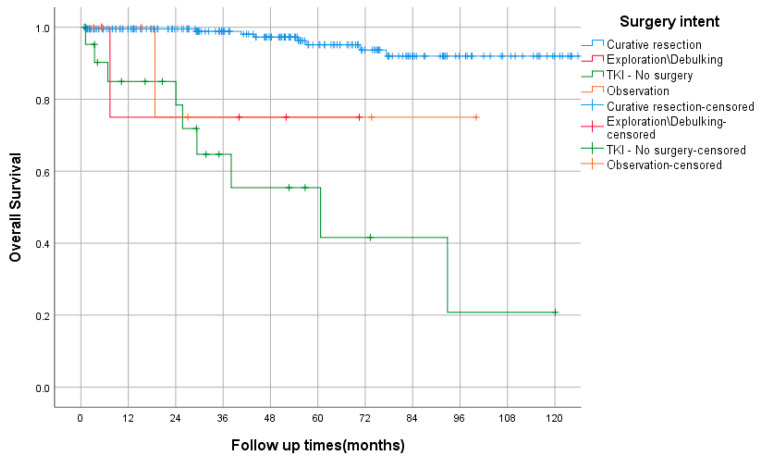
Overall survival based on management approach using Kaplan–Meier curve.

The OS based on management approach is shown in Figure 1. In total, 86% (*n* = 213) underwent curative surgical resection, including eight patients with advanced/metastatic disease. Forty-nine patients received adjuvant imatinib. Among the NIH intermediate and high-risk groups (90 patients), only 40 patients (44%) received adjuvant imatinib, and only 15/40 (38%) completed three years of adjuvant imatinib. Seventeen patients (7%) had recurrence after curative treatment, including adjuvant TKI. Thirteen patients were treated with neoadjuvant imatinib. Forty-three patients (17%) received at least one line of palliative TKI. Seven patients received three lines of TKI. TKI use is shown in Table 3. 

### 3.8. Survival 

**Figure 2:**
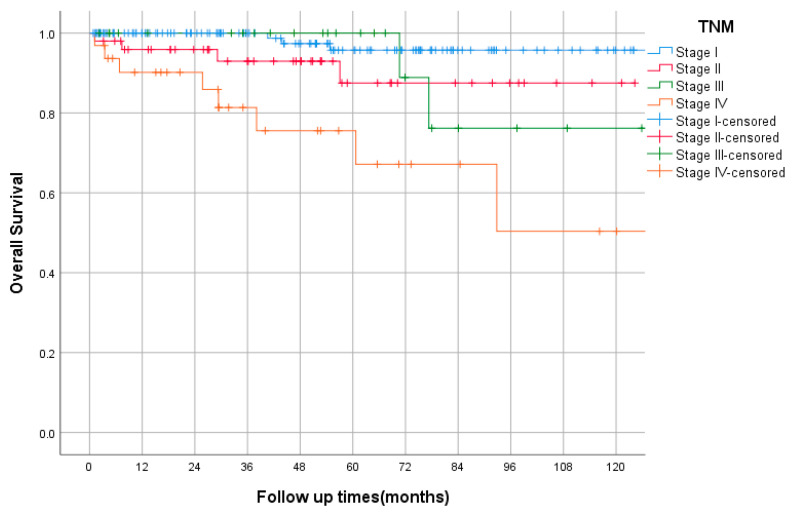
Overall survival by TNM stage using Kaplan–Meier curve.

**Figure 3:**
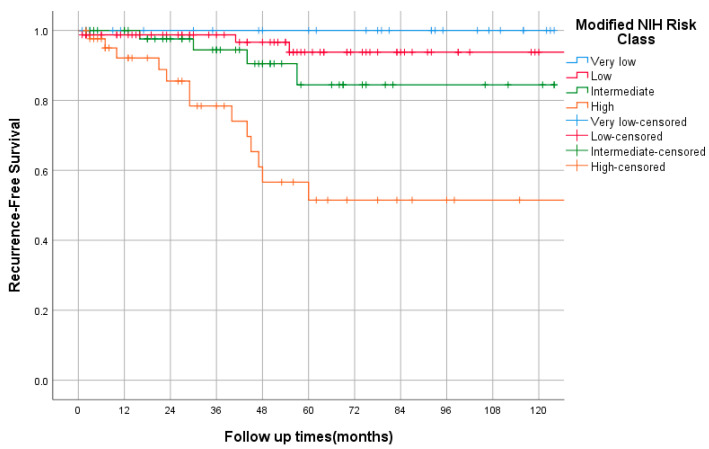
Recurrence-free survival by NIH risk class using Kaplan–Meier curve.

The median follow-up was 47 months (range 1–137). The OS, by TNM stage, is shown in Figure 2. In the entire study cohort, death was documented in 18 cases (8%). The 5-yearsrecurrence-free survival (RFS) rates for very low and low risk were 100% and 94%, respectively, while those for intermediate and high risks were 84% and 51%, respectively, as shown in Figure 3. Among the intermediate/high-risk group, patients who did not receive adjuvant imatinib had longer RFS than those who received adjuvant IM with 5-year RFS rates of 89% and 54%, respectively, *p* = 0.059, HR = 0.34 (95% CI, 0.11–1.04). The median PFS of first-line imatinib was 40 months (95% CI, 12 to 69 months). The 2- and 5-year PFS rates were 51% and 39%, respectively. The 5-year overall survival (OS) rate was 100%, that of very low and low risk was 94%, while those of intermediate, high risk, and advanced were 91%, 88%, and 65%, respectively. Using the Kaplan–Meier method, there were statistically significant differences in RFS and OS between NIH risk groups, both *p* < 0.0005. 

In univariate analysis, ECOG at diagnosis, site, mitosis, secondary malignancy, and size were significant predictors for OS. By multivariate analysis, poor performance status (ECOG 3) was a predictor for shorter OS (*p* < 0.005). The other factors analyzed (patient gender, site, histology, size, mitosis, and presence of secondary malignancy) were insignificant. In univariate and multivariate analysis, high mitosis and large size (>5 cm) were associated with worse RFS, for both *p* < 0.002 and *p* < 0.016, respectively. 

## 4. Discussion

Although many clinical trials evaluate the various treatment options for GIST, available documentation of the outcomes in the real-world data from Canada is limited. The present study explores the outcomes and clinicopathological features of 248 GIST cases treated at the Ottawa Hospital over the past 10 years. In our study, the most common GIST locations were the stomach (63%) and small bowel (29%). Nearly 35% of patients were asymptomatic at the time of diagnosis, and their tumors were found incidentally. This finding is comparable to other reports and could be explained by the small size of the tumors, less than 5 cm, and the indolent course of these tumors [18]. Although males and females were equally distributed, males have a more aggressive and higher-risk disease. In contrast, an Asian study reported higher recurrence or metastasis in females than males [19]. Around 13% presented with de novo metastasis, similar to a study from British Columbia [20].

Unlike solid organ tumors, the behavior of GIST is variable, though it is always malignant if it measures more than 2 cm. Therefore, risk assessment tools were developed to predict malignant potential [21]. Modified NIH and Miettinen criteria are commonly used assessment tools. In this study, nearly 68% and 54% had no or low-risk disease, respectively, using Miettinen and NIH criteria. This is comparable with the described risk classes in other studies [22,23]. There are limited data about the optimal risk assessment tool; however, we observed overlapping between low and moderate risk using Miettinen criteria, while NIH appeared more accurate in predicting the outcome. Epithelioid histology looked to have a more favorable prognosis than spindle cell or mixed histology. However, there is no statistically significant association between type of histology and risk of recurrence (*p* = 0.41).

Surgical resection remains the only curative intervention for localized GIST tumors. Patients with bulky or limited metastases could benefit from surgical management. In this study, around 86% had curative surgical resection with a 5-year survival rate of 94%. This is significantly better than the outcome of surgery in the pre-imatinib era (5-year OS of ~54%) and comparable to reported data in the imatinib era [23,24].

Adjuvant imatinib has substantially improved recurrence-free survival in many phase III trials, particularly in intermediate and high-risk groups. The Scandinavian Sarcoma Group (SSG XVIII/AIO) trial is a phase III trial that compared 36 vs. 12 months of adjuvant imatinib and showed significant improvement in the 5-year RFS and OS rates in patients who received 36 months of adjuvant imatinib compared to 12 months. The 5-year RFS rates were 71.1% vs. 52.3%, respectively; *p* < 0.001; the 5-year OS rates were 91.9% vs. 85.3%, respectively; *p* = 0.036. A recent update showed that the 10-year RFS rates were 52.5% vs. 41.8%, respectively. Further exploratory analysis showed that patients with KIT exon 11 deletion mutations benefit most from the longer duration of adjuvant imatinib [7,8,25,26].

In our study, the 5-year RFS rate for intermediate and high risk were 84% and 51%, respectively, comparable to the clinical trials. However, 56% (50/90) did not receive adjuvant imatinib. Interestingly, by comparing subgroups, we found that patients who received adjuvant IM had a trend toward shorter RFS; however, HR was not significant. Although there is no clear explanation, possible reasons could be the relatively small sample size; shorter adjuvant duration, as only 15/40 (38%) completed three years of adjuvant imatinib; and more inclusion of intermediate risk (35%, 14) in the adjuvant group. At the same time, the no-adjuvant group has a predominantly intermediate risk (68%, 34), a lack of mutational testing, and more deaths than the adjuvant group. Moreover, there was no statistically significant difference in OS between those who did or did not receive adjuvant imatinib, with a *p*-value of 0.151. This is also similar to the COSOG Z9001 study, a phase III trial that evaluated imatinib 400 mg vs. a placebo for one year, as adjuvant treatment, which did not show OS benefit. Moreover, this emphasizes the importance of 3 years of adjuvant imatinib. Less than three years of adjuvant imatinib were evaluated in the EORTC-62024 study: the 5-year imatinib failure-free survival (IFFS) rate did not reach significance, at 87% vs. 84% (HR, 0.79; 98.5% CI, 0.50–1.25; *p* = 0.21). 

Unresectable or metastatic KIT-positive GISTs are common, and imatinib is considered the standard first-line treatment. The Intergroup S0033 study evaluated two doses of imatinib (400 mg daily) vs. a high dose (800 mg) and found that the median OS rates were 55 and 51 months, respectively, after a median follow-up of 4.5 years. The high-dose arm had more grade 3, 4, and 5 toxicities [9]. In this study, the median PFS of first-line imatinib was 40 months (95%CI, 12 to 69 months). The 2- and 5-year PFS rates were 51% and 39%, respectively. These are slightly better than the data reported in the Intergroup S0033 study. Blanke CD et al. reported a 5-year survival rate of 55%, in patients with advanced GIST who were treated with imatinib, regardless of a 400 or 600 mg/d starting dose [27]. In contrast, this study showed that the 5-year overall survival (OS) rate was 65% for the same group of patients; this could be explained by the availability of other TKI options after imatinib failure.

Treatment options for GIST are expanding with evolving sequencing technology. There is growing evidence of the cost-effectiveness of precision-medicine-assisted imatinib treatment, compared with empirical treatment. T. Patterson et al. conducted a population-based study in British Columbia. They found that mutational analysis (MA) was ordered in 41% of patients and MA use increased after 2015, especially in the metastatic setting. In our hospital, MA is not routinely performed; however, despite limited use of MA, OS remains comparable to the reported literature [20,28]. 

Our study has limitations, including the retrospective design and lack of MA data. It could not collect the complete toxicity data of TKI. Other limitations include the relatively limited sample size, especially for patients who received TKI. 

## 5. Conclusions

Curative surgical resection remains the gold standard management of GIST. Our results are comparable to the reported literature. Further research is needed to explore histology’s role in risk stratification and initiating adjuvant TKI.

## Figures and Tables

**Table 1:** Baseline patients’ characteristics.

	Total *n* = 248 (Percentage %)		
**Age**	Median	64 (Range 28–90)	
**Gender**	Male	124	50%
	Female	124	50%
**Comorbidities**	HTN	103	41.5%
	DM	49	19.8%
	DLP	75	30.2%
	GERD	25	10%
	IHD	28	11.3%
	Neurofibromatosis	4	1.6%
	Other	104	42%
**Clinical presentation**	Incidental	87 *	35%
	Abdominal pain	84	34%
	GI bleeding	59	24%
	Anemia	48	19%
	Bowel obstruction	7	2.8%
	Perforation	3	1.2%
	Other	56	23%
Duration of symptom **	<14 days	41	16.5%
	>14 day	32	12.9%
	>2 months	38	15.3%
	>6 months	32	12.9%
	NA	24	9.7%
**ECOG**	0	100	40.3%
	1	77	31%
	2	7	2.8%
	3	4	1.6%
	NA	22	8.9%
**Mode of initial diagnosis**	CT	123	49.5%
	Endoscopy	63	25%
	Surgical exploration	14	5.6%
	EUS	13	5.2%
	MRI	10	4%
	Other	11	4%
**Curative surgical resection**		213	85.9%

* Eighty-one patients were lacking symptoms, while six patients presented with non-specific abdominal pain (e.g., flank pain). ** not including incidental diagnosis. HTN: hypertension, DM: diabetes mellitus, DLP: dyslipidemia, GERD: gastroesophageal reflux disease, IHD: ischemic heart disease, ECOG: Eastern Cooperative Oncology Group performance status, CT: computed tomography, EUS: endoscopic ultrasound, MRI: magnetic resonance imaging.

**Table 2:** GIST sites and the pathology data.

	Total *n* = 248 (Percentage %)		
**Site**	Esophagus	1	0.4%
	Stomach	156	62.9%
	Small bowel	73	29.4%
	Duodenum	19	7.7%
	Jejunum	19	7.7%
	Ileum	13	5.2%
	Unspecified	22	8.9%
	Appendix	1	0.4%
	Colon	2	0.8%
	Rectum	5	2%
	Diffuse/overlapping	8	3.2%
	Mesentery	2	0.8%
**Miettinen** **risk class**	None	23	11%
	None–rare **	5	2%
	Very low	44	22%
	Low	68	33%
	Moderate	39	19%
	High	24	12%
**Modified NIH risk class**	Very low	29	14%
	Not defined **	5	2%
	Low	78	38%
	Intermediate	48	24%
	High	42	21%
**Histology**	Spindle cell	159	64.1%
	Epithelioid	18	7.3%
	Mixed	39	15.7%
	NA *	32	12.9%
**Size**	<2 cm	37	14.9%
	2–5	91	36.7%
	5–10	70	28.2%
	>10	31	12.5%
	NA	19	7.7%
**Mitosis**	≤5 hpf	177	71.4%
	>5 hpf	45	18.1%
	NA	26	10.5%
**Grade**	1	159	64.1%
	2	36	14.5%
	3	5	2%
	Unknown	48	19.4%
**Mutation**	No study	237	95.6%
	Study	11	4.4%
	KIT Exon 11	3	1.2%
	PDGFR Exon 18	4	1.6%
	PDGFR c.2525A	1	0.4%
	SDHB	2	0.8%
	Other	1	0.4%
**TNM stage**	I	131	52.8%
	II	50	20.2%
	III	25	10.1%
	IV	32	12.9%
	NA	10	4%

* NA: no available data. ** This category not defined clearly in Miettinen or NIH criteria (size ≤ 2 cm, and mitotic rate > 5 per 50 HPFs).

**Table 3:** Utilization of TKI in the study population.

	Total *n* = 248 (Percentage %)		
**Adjuvant Imatinib**		49 *	19.8
**Neoadjuvant Imatinib**		13	5.2%
**Palliative TKI**	Imatinib	41	16.5%
	Sunitinib	13	5.2%
	Regorafenib	7	2.8%
	Ripertinib	3	1.2%
	TKI alone (no surgery)	22	8.9%

* Forty patients had intermediate or high-risk disease, while three patients had low risk. Four patients had resected metastatic disease and two patients had unknown risk.

## Data Availability

The data that support the findings of this study are available from the corresponding author upon reasonable request.
